# Risk of cardiovascular disease in lean patients with nonalcoholic fatty liver disease

**DOI:** 10.1186/s12876-023-02848-7

**Published:** 2023-06-17

**Authors:** Shun Ishido, Nobuharu Tamaki, Yuka Takahashi, Naoki Uchihara, Keito Suzuki, Yuki Tanaka, Haruka Miyamoto, Michiko Yamada, Hiroaki Matsumoto, Tsubasa Nobusawa, Taisei Keitoku, Kenta Takaura, Shohei Tanaka, Chiaki Maeyashiki, Yutaka Yasui, Kaoru Tsuchiya, Hiroyuki Nakanishi, Masayuki Kurosaki, Namiki Izumi

**Affiliations:** Department of Gastroenterology and Hepatology, Musashino Red Cross Hospital, 1-26-1 Kyonan-Cho, Musashino-Shi, Tokyo, 180-8610 Japan

**Keywords:** NAFLD, Lean NAFLD, Non-lean NAFLD, Cardiovascular disease

## Abstract

**Background:**

Patients with nonalcoholic fatty liver disease (NAFLD) are highly at risk for cardiovascular disease (CVD). However, the risk of developing CVD in patients with lean NAFLD is not yet fully understood. Therefore, this study aimed to compare the CVD incidence in Japanese patients with lean NAFLD and those with non-lean NAFLD.

**Methods:**

A total of 581 patients with NAFLD (219 with lean and 362 with non-lean NAFLD) were recruited. All patients underwent annual health checkups for at least 3 years, and CVD incidence was investigated during follow-up. The *primary end-point* was CVD incidence at 3 years.

**Results:**

The 3-year new CVD incidence rates in patients with lean and non-lean NAFLD were 2.3% and 3.9%, respectively, and there was no significant difference between two groups (*p* = 0.3). Multivariable analysis adjusted for age, sex, hypertension, diabetes, and lean NAFLD/non-lean NAFLD revealed that age (every 10 years) as an independent factor associated with CVD incidence with an odds ratio (OR) of 2.0 (95% confidence interval [CI]: 1.3–3.4), whereas lean NAFLD was not associated with CVD incidence (OR: 0.6; 95% CI: 0.2–1.9).

**Conclusions:**

CVD incidence was comparable between patients with lean NAFLD and those with non-lean NAFLD. Therefore, CVD prevention is needed even in patients with lean NAFLD.

## Background

Nonalcoholic fatty liver disease (NAFLD) currently occurs in > 25% of the world population and is one of the major health problems [1–5]. Patients with NAFLD have high risk for developing liver-related events including hepatocellular carcinoma and decompensation. Prevention and early detection of these complications are important clinical issue [6–10]. Because NAFLD is a disease associated with metabolic syndrome and is frequently complicated by diabetes and hyperlipidemia [11–13], patients with NAFLD are also highly at risk for cardiovascular disease (CVD) events [14, 15]. CVD has been known to more frequently occur in patients with NAFLD than in the general population [16]. Therefore, CVD prevention is an important issue that should be considered in patients with NAFLD [17–20]. Because NAFLD is strongly associated with metabolic syndrome, many patients with NAFLD are obese. However, some patients have lean NAFLD (defined as a BMI of < 25 kg/m^2^ in Western subjects and < 23 kg/m^2^ in Asian subjects) [21]. In addition, whether lean NAFLD is associated with disease progression, especially the risk of developing CVD, is not yet fully understood. Furthermore, the rate of lean NAFLD varies by race and region [22]. Therefore, even among patients with lean NAFLD, the risk of CVD may also differ by race and region, and the association between each of these factors and CVD risk remains to be verified. Therefore, this study aimed to examine whether CVD incidence differs between lean and non-lean NAFLD in Japanese patients.

## Methods

### Study protocol

**Fig. 1:**
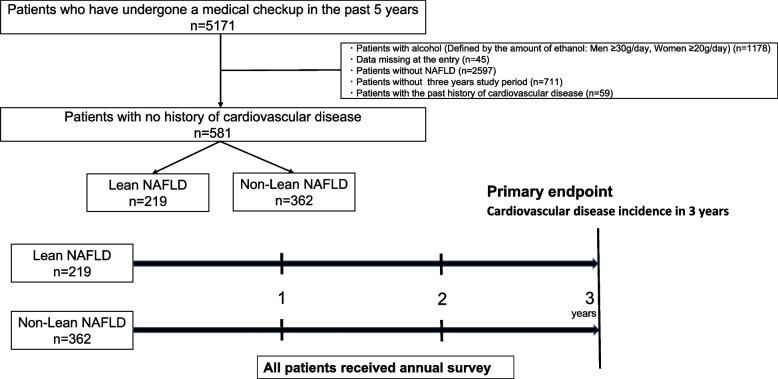
Study flow chart

This is a single-center, retrospective study of 5171 patients who underwent health checkup at Musashino Red Cross Hospital from January 2017 to May 2022. A total of 1351 patients with NAFLD were included, excluding 1178 with a history of alcohol consumption (defined as daily alcohol consumption of at least 30 g/day of ethanol for men and 20 g/day for women [23]), 2597 without fatty liver, and 45 with missing data. Patients underwent annual health checkups at least 3 years. After excluding 711 patients who did not reach a 3-year follow-up and 59 with a history of CVD, 581 patients were finally analyzed: 219 patients with lean NAFLD (BMI < 23 kg/m^2^) and 362 patients with non-lean NAFLD (BMI ≥ 23 kg/m^2^) (Fig. 1). This study was conducted following the principles of the Declaration of Helsinki and with the consent of the ethics committee of the institution where the study was conducted (Approval Number: 1005). We obtained consent from all patients using an opt-out approach.

### Clinical and laboratory data

Age, sex, weight, smoking history, alcohol intake, and blood test data were recorded on the date of the first medical checkup.

### Definition of fatty liver

Ultrasonographic findings of parenchymal brightness, liver-to-kidney contrast, deep beam attenuation, and bright vessel walls were considered indicators of a fatty liver at the first medical checkup [24, 25].

### Definition of comorbidity status

Diabetes mellitus was defined as a fasting plasma glucose level of ≥ 126 mg/dL, hemoglobin A1c (HbA1c) level of ≥ 6.5%, or use of any antihyperglycemic medication [26]. Dyslipidemia was defined as elevated triglycerides (≥ 150 mg/dL), elevated low-density lipoprotein (≥ 140 mg/dL), decreased high-density lipoprotein (< 40 mg/dL in men and < 50 mg/dL in women), or use of any lipid-lowering medication [27]. Hypertension was defined as systolic blood pressure of ≥ 140 mmHg, diastolic blood pressure of ≥ 90 mmHg, or use of any antihypertensive medication [28]. Significant alcohol consumption was defined as > 30 and > 20 g/week in men and women, respectively [23].

### Diagnosis of CVD

CVD was defined as ischemic heart disease, heart failure, cerebrovascular disease, and peripheral arterial disease. Its incidence was investigated through interviews at the time of annual health checkups or from medical records.

### Primary outcome

The primary outcome was the occurrence of new cardiovascular events within 3 years, and the incidence of cardiovascular events in patients with lean and non-lean NAFLD was compared.

### Statistical analysis

Patient backgrounds of lean and non-lean NAFLD were compared using Student’s t-test or Fisher’s exact test. Logistic analysis was used to compare factors associated with new CVD events over 3 years. The logistic analysis included the following factors: age, gender, diabetes, hypertension, dyslipidemia as well as lean or non-lean NAFLD. Of these metabolic associated factors (diabetes, hypertension, and dyslipidemia), factors that were significant in univariate analysis were entered into the multivariate analysis. *P*-values of < 0.05 were considered statistically significant. All statistical analyses were performed using EZR [29].

## Results

### Patient characteristics

The numbers of patients with lean and non-lean NAFLD were 219 and 362, respectively. The mean ± standard deviation values of the patients’ age were 58 ± 12 and 59 ± 11 years, respectively, without significant differences between the two groups (*p* = 0.8). A total of 110 (50.2%) and 220 (60.8%) males had lean and non-lean NAFLD, respectively (*p* = 0.02); 47 (21.5%) and 138 (38.1%) had hypertension (*p* < 0.01); and 20 (9.1%) and 57 (15.7%) had diabetes (*p* = 0.02). In the non-lean NAFLD group, rates of men, hypertension, and diabetes were significantly higher than the lean-NAFLD group. Dyslipidemia was observed in 108 (49.3%) and 201 (55.5%) (*p* = 0.2) and smoking history was observed in 23 (10.5%) and 44 (12.2%) patients (*p* = 0.6; Table 1) in the lean and non-lean NAFLD groups, respectively. The median (interquartile range) systolic and diastolic blood pressure of patients with hypertension was 128 (118–142) and was 81 (74–87] mmHg, respectively. HbA1c of patients with diabetes was 6.8 (6.5–7.2] % and low-density lipoprotein cholesterol of patients with dyslipidemia was 143 (110–158) mg/dL.

**Table 1:** Characteristics of patients with NAFLD

	Lean *n* = 219	Non-Lean *n* = 362	*P* Value
Age, years	58 ± 12	59 ± 11	*p* = 0.8
Gender, male (%)	110 (50.2%)	220 (60.8%)	*p* = 0.02
BMI, kg/m^2^	21.5 ± 1.1	26.2 ± 2.8	*p* < 0.01
Smoking	23(10.5%)	44 (12.2%)	*p* = 0.6
Dyslipidemia	108 (49.3%)	201 (55.5%)	*p* = 0.2
Hypertension	47 (21.5%)	138 (38.1%)	*p* < 0.01
Diabetes	20 (9.1%)	57 (15.7%)	*p* = 0.02
AST, IU/L	22 ± 6	24 ± 8	*p* < 0.01
ALT, IU/L	23 ± 11	27 ± 13	*p* < 0.01
Albumin, g/dL	4.3 ± 0.3	4.3 ± 0.2	*p* = 0.4
GGT, IU/L	30 ± 22	39 ± 34	*p* < 0.01
Platelet counts, 10^9^/L	237 ± 55	236 ± 61	*p* = 0.8
Total cholesterol, mg/dL	205 ± 33	204 ± 33	*p* = 0.8
Triglycerides, mg/dL	104 ± 64	127 ± 71	*p* < 0.01
HDL, mg/dL	64 ± 15	58 ± 14	*p* < 0.01
LDL, mg/dL	124 ± 27	126 ± 29	*p* = 0.5

Continuous data are shown in the mean ± standard deviation (SD). *P* value indicates difference between Lean and Non-Lean

*Abbreviations*: *BMI* body mass index, *ALT* alanine aminotransferase, *AST* aspartate aminotransferase, *GGT* gamma-glutamyl transpeptidase, *HDL* high-density lipoprotein, *LDL* low-density lipoprotein

### The percentage of new CVD incidence in 3 years

**Fig. 2:**
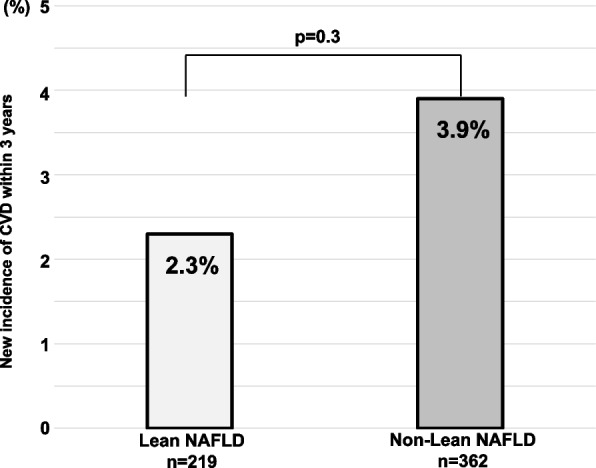
The percentage of new incident CVD in 3 years

The 3-year incidence rates of new CVD in the lean and non-lean NAFLD groups were 2.3% and 3.9%, respectively, without a significant difference (*p* = 0.3, Fig. 2). In the lean NAFLD group, 5 of 219 patients developed CVD: 4 cerebral infarctions and 1 myocardial infarction. In the non-lean NAFLD group, 14 of 362 patients developed CVD: 4 cerebral infarctions, 2 transit ischemic attacks, 2 heart failure, 4 myocardial infarctions, and 2 peripheral artery diseases.

### Factors of new CVD incidence in 3 years

Factors associated with the development of new CVD were examined. Multivariate analysis revealed that age (in every 10 years) (odds ratio [OR]: 2.0; 95% confidence interval [CI]: 1.3–3.4; *p* < 0.01) was an independent factor associated with the development of new CVD, whereas lean NAFLD (OR: 0.6; 95% CI: 0.2–1.9; *p* = 0.4) was not associated with CVD development (Table 2).

**Table 2:** Factors of new cardiovascular disease incidence with NAFLD in 3 years

	Univariable OR (95% CI)	*P* Value	Multivariable OR (95% CI)	*P* Value
Age (every 10 years)	2.2 (1.4–3.4)	*p* < 0.01	2.0 (1.3–3.4)	*p* < 0.01
Gender, male (%)	1.3 (0.5–3.4)	*p* = 0.6	1.0 (0.4–2.9)	*p* = 0.9
Lean	0.6 (0.2–1.6)	*p* = 0.3	0.6 (0.2–1.9)	*p* = 0.4
Hypertension	3.9 (1.5–9.9)	*p*<0.01	2.6 (0.9–7.4)	*p* = 0.07
Dyslipidemia	0.3 (0.1–1.2)	*p* = 0.2		
Diabetes	3.2 (1.2–8.7)	*p* = 0.02	1.6 (0.5–4.8)	*p* = 0.4

## Discussion

### Primary findings

This study found that lean NAFLD had a similar CVD risk as non-lean NAFLD. Patients with lean NAFLD had a lower prevalence of diabetes and hypertension compared to patients with non-lean NAFLD. However, after adjusting for these factors, no difference was observed in the CVD risk between lean and non-lean NAFLD. Therefore, lean NAFLD should be treated with the same caution for CVD development as non-lean NAFLD.

### In context with published literature

The significance of disease progression in lean NAFLD is not yet fully clarified. In particular, reports comparing the CVD incidence between lean and non-lean NAFLD are limited. A study that primarily included Hispanics (40.8% of whom had NAFLD without obesity) in the United States reported no significant difference in the incidence of new CVD between the lean and non-lean NAFLD groups [30]. Conversely, a study that included mainly Caucasians (approximately 10% of participants had lean NAFLD) in the United States revealed that lean NAFLD has a lower incidence of CVD compared to non-lean NAFLD [31]. Another study that included mainly Asian in China showed that the CVD risk was lower in lean NAFLD than in non-lean NAFLD [32]. Thus, whether CVD risk was higher in lean NAFLD than in non-lean NAFLD remains unclear. Furthermore, genetic polymorphisms such as PNPLA3 are associated with the development of lean NAFLD [33–35]. Because these genetic polymorphisms vary by race, the prevalence of lean NAFLD also varies by race. The prevalence of lean NAFLD varies from 7% in the United States [36] to 19% in Asia [37, 38]. Therefore, racial differences may affect the CVD risk in lean NAFLD and regional validation is needed. In this study, the proportion of lean NAFLD was 37.6%, which is higher than that in other studies. In this situation, this study revealed that lean NAFLD has the same CVD risk as non-lean NAFLD. The results provide further insight into the risk of CVD in patients with lean NAFLD.

The prevalence of dyslipidemia, hypertension and diabetes are higher in patients with non-lean NAFLD. In several previous studies demonstrated that patients with lean NAFLD had a similar incidence of CVD compared to non-lean NAFLD [30, 39]. Focusing on patient characteristics, diabetes, dyslipidemia, and hypertension were well controlled even in patients with non-lean NAFLD and it may affect the less impact on the CVD incidence.

### Strengths and limitations

The strength of this study is that all patients received annual health checkup with the uniform protocol, which enabled an accurate assessment of new CVD development. In this study, the proportion of lean NAFLD was 37.6%, which is higher than that in other studies. In a previous study that investigated the epidemiology of NAFLD in Japan with a focus on lean NAFLD, the proportion of patients with lean NAFLD in Japan was 20.7%, which was lower than in our study [40]. Patient characteristics were similar between the previous study and this study. One reason for the difference in the prevalence of lean NAFLD is that this study was conducted in subjects with health check-up with at least more than 3 years of follow-up and the cohort is not a hospital or population-based cohort. Therefore, further validation studies with a hospital or population-based cohort are needed. This study was conducted at a single institution (in Japan only), there were a limited number of cases, and the results were only available for 3 years. Thus, the results may differ with more cases or longer-term observation.

### Future implications

NAFLD is a risk factor for hepatocellular carcinoma and decompensation as well as CVD [41–43]. However, the risk of CVD in lean NAFLD has not been fully elucidated. Our study shows that the CVD risk in lean NAFLD is as high as in non-lean NAFLD, indicating that even lean NAFLD should be considered a risk factor for CVD. The study was conducted in a population with a high proportion of lean NAFLD and the results provide a new insight associated with CVD risk in patients with lean NAFLD. These results provide important guidelines for the treatment of NAFLD. Recently, the new concept of metabolic dysfunction-associated fatty liver disease (MAFLD) has been proposed to detect high risk patients for complications. A previous study demonstrated that MAFLD better identified patients with worsening of CVD risk than NAFLD [14]. Investigation for the association between MAFLD and CVD incidence is also needed in a future study.

## Conclusions

In conclusion, CVD incidence was comparable between patients with lean NAFLD and those with non-lean NAFLD. Therefore, CVD prevention is needed even in patients with lean NAFLD.

## Data Availability

The datasets generated and analyzed during the current study are not publicly available due (because the datasets have personal information) but are available from the corresponding author on reasonable request.

## Abbreviations

NAFLD: Nonalcoholic fatty liver disease
CVD: Cardiovascular disease
BMI: Body mass index
ALT: Alanine aminotransferase
AST: Aspartate aminotransferase
GGT: Gamma-glutamyl transpeptidase
HDL: High-density lipoprotein
MAFLD: Metabolic dysfunction-associated fatty liver disease
LDL: Low-density lipoprotein
